# A comparative PET imaging study with the reversible and irreversible EGFR tyrosine kinase inhibitors [^11^C]erlotinib and [^18^F]afatinib in lung cancer-bearing mice

**DOI:** 10.1186/s13550-015-0088-0

**Published:** 2015-03-20

**Authors:** Paul Slobbe, Albert D Windhorst, Marijke Stigter-van Walsum, Egbert F Smit, Heiko G Niessen, Flavio Solca, Gerd Stehle, Guus A M S van Dongen, Alex J Poot

**Affiliations:** Department of Radiology and Nuclear Medicine, VU University Medical Center, De Boelelaan 1117, Amsterdam, 1081 HV The Netherlands; Department of Otolaryngology/Head and Neck Surgery, VU University Medical Center, De Boelelaan 1117, Amsterdam, 1081 HV The Netherlands; Department of Pulmonary Diseases, VU University Medical Center, De Boelelaan 1117, Amsterdam, 1081 HV The Netherlands; Department of Translational Medicine and Clinical Pharmacology, Boehringer Ingelheim Pharma GmbH and Co. KG, Birkendorfer Straße 65, 88397 Biberach an der Riss, Germany; Department of Pharmacology and Translational Research, Boehringer Ingelheim GmbH and Co. KG, Doktor-Boehringer-Gasse 5-11, Vienna, A-1120 Austria; Therapeutic Area Oncology, Boehringer Ingelheim Pharma GmbH and Co. KG, Birkendorfer Straße 65, 88397 Biberach an der Riss, Germany

**Keywords:** Afatinib, Erlotinib, Activated EGFR, Radiochemistry, PET, Personalized medicine

## Abstract

**Background:**

Tyrosine kinase inhibitors (TKIs) have experienced a tremendous boost in the last decade, where more than 15 small molecule TKIs have been approved by the FDA. Unfortunately, despite their promising clinical successes, a large portion of patients remain unresponsive to these targeted drugs. For non-small cell lung cancer (NSCLC), the effectiveness of TKIs is dependent on the mutational status of epidermal growth factor receptor (EGFR). The exon 19 deletion as well as the L858R point mutation lead to excellent sensitivity to TKIs such as erlotinib and gefitinib; however, despite initial good response, most patients invariably develop resistance against these first-generation reversible TKIs, e.g., via T790M point mutation. Second-generation TKIs that irreversibly bind to EGFR wild-type and mutant isoforms have therefore been developed and one of these candidates, afatinib, has now reached the market. Whether irreversible TKIs differ from reversible TKIs in their *in vivo* tumor-targeting properties is, however, not known and is the subject of the present study.

**Methods:**

Erlotinib was labeled with carbon-11 and afatinib with fluorine-18 without modifying the structure of these compounds. A preclinical positron emission tomography (PET) study was performed in mice bearing NSCLC xenografts with a representative panel of mutations: an EGFR-WT xenograft cell line (A549), an acquired treatment-resistant L858R/T790M mutant (H1975), and a treatment-sensitive exon 19 deleted mutant (HCC827). PET imaging was performed in these xenografts with both tracers. Additionally, the effect of drug efflux transporter permeability glycoprotein (P-gp) on the tumor uptake of tracers was explored by therapeutic blocking with tariquidar.

**Results:**

Both tracers only demonstrated selective tumor uptake in the HCC827 xenograft line (tumor-to-background ratio, [^11^C]erlotinib 1.9 ± 0.5 and [^18^F]afatinib 2.3 ± 0.4), thereby showing the ability to distinguish sensitizing mutations *in vivo*. No major differences were observed in the kinetics of the reversible and the irreversible tracers in each of the xenograft models. Under P-gp blocking conditions, no significant changes in tumor-to-background ratio were observed; however, [^18^F]afatinib demonstrated better tumor retention in all xenograft models.

**Conclusions:**

TKI-PET provides a method to image sensitizing mutations and can be a valuable tool to compare the distinguished targeting properties of TKIs *in vivo*.

## Background

Recent developments in molecular biology have led to an increased understanding of the signal transduction pathways in cancer, and crucial molecular targets have been identified that are involved in cancer growth, survival, and metastasis. Furthermore, increased structural understanding of proteins and their specific chemical interactions combined with high throughput screening and medicinal chemistry efforts has led to major breakthroughs in drug discovery. Together, this has led to the development of tailor-made targeted pharmaceuticals as anti-cancer drugs. Receptor tyrosine kinases (RTKs) form a family of transmembrane proteins that have received a lot of interest as they play a pivotal role in the signal transduction pathways of the cell. RTKs consist of an extracellular domain capable of ligand binding and an intracellular domain for downstream signaling. Prominent members of this family include the epidermal growth factor receptor (EGFR) and the vascular endothelial growth factor receptor (VEGFR) [1,2].

The development of small molecules targeting kinases has expanded enormously in the last decade. Over 15 small molecule tyrosine kinase inhibitors (TKIs) have been approved by the US Food and Drug Administration with an estimated several hundreds under (pre)clinical development. These TKIs act on the intracellular catalytic kinase domain by competing with ATP and induce inhibition of downstream signaling [3]. Good cell penetration and long-lasting, high-affinity binding to the RTK are required to effectively compete with the high intracellular ATP concentration [4]. Although the approval rate of new TKIs is high and substantial patient benefit is achieved, there is a lack of long-term efficacy in certain patients with RTK-driven tumors. The underlying cause of this inter-patient variability is best understood for EGFR-targeting kinase inhibitors in non-small cell lung cancer (NSCLC) [5].

Activating mutations in the kinase domain of EGFR dictate effectiveness of TKIs that are currently on the market. The most common sensitizing mutations are small in-frame deletions in exon 19 (45%) or the L858R missense mutation (40% to 45%) in exon 21 leading to favorable response rates to EGFR TKIs [6]. As a result, EGFR TKI therapy is especially effective in NSCLC patients with tumors displaying an activating EGFR mutation which occurs in 5% to 25% of the Caucasian NSCLC patient population [7,8]. In clinical practice, mutational status is determined by an invasive biopsy of the tumor tissue, which does not always provide a representative overview of the genomic heterogeneity of the tumor [9]. Unfortunately, despite initial promising response, most patients develop resistance against first-generation reversible TKIs such as erlotinib and gefitinib [10]. About half of the recurrences are associated with the occurrence of an additional point mutation, i.e., the exon 20 T790M, which compared to the single EGFR mutant, displays increased affinity for ATP and thus reduced affinity for first-generation reversible inhibitors.

Second-generation TKIs such as dacomitinib or afatinib that covalently bind to EGFR have been developed, and afatinib has recently obtained marketing approval for first-line treatment of lung cancer patients with common activating EGFR mutations [11]. This new generation of inhibitors not only binds covalently to their target molecules but also inhibits all kinase-competent members of the ErbB receptor family, which are EGFR, HER2, and ERBB4. Most mutant isoforms of these ErbB receptors including EGFR T790M are also inhibited by these new molecules, which therefore bear the potential to delay or even circumvent some of the resistance mechanisms set off by first-generation inhibitors [1].

In recent publications we, among others, have demonstrated the use positron emission tomography (PET) imaging with radiolabeled TKIs (TKI-PET) as a tool to address TKI disposition *in vivo*. By labeling the TKI with a positron emitting radionuclide and maintaining its original structure, these PET tracers can be used to assess the *in vivo* biodistribution, pharmacokinetics (at tracer level), off-target binding, and more importantly tumor targeting of the therapeutic itself by means of PET [12,13]. TKI-PET could also become a technique to identify patients who might benefit from treatment, thus providing a non-invasive predictive tool for personalized medicine [3,12,13]. However, whether irreversible TKIs differ from reversible TKIs in their *in vivo* tumor targeting properties, is not known and is subject of this study.

**Figure 1:**
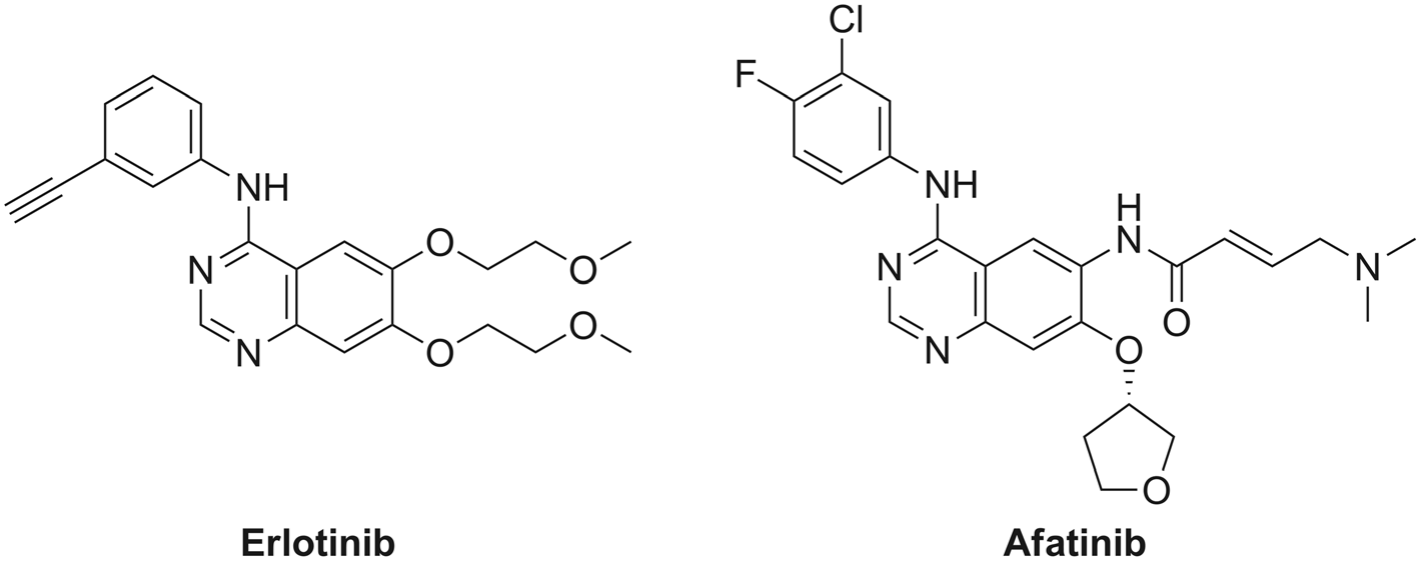
**Chemical structures of erlotinib and afatinib.**

Erlotinib (Tarceva*®*, Roche, Basel, Switzerland; **1**, Figure 1) is a first-generation reversible 4-anilinoquinazoline inhibitor of EGFR and was approved in 2004 for NSCLC treatment of patients with locally advanced or metastatic NSCLC in combination with chemotherapy. No patient selection based on mutation analysis was performed at the time of approval and all eligible NSCLC patients were treated with erlotinib [14]. In the following years, it became clear that EGFR mutations are of great importance for the efficacy of erlotinib (and other first-generation EGFR TKIs, such as gefitinib), and erlotinib was subsequently approved in 2013 as a first-line treatment for patients whose tumors harbor an exon 19 deletion or an exon 20 point mutation [15]. TKI-PET imaging with the tracer [^11^C]erlotinib was first reported by Memon et al. in a seminal study demonstrating selective uptake in treatment-sensitive xenografts with mutation-activated EGFR. In this pre-clinical study, no metabolite analysis was performed [16]. Subsequently, a clinical evaluation was performed by the same group in NSCLC patients demonstrating uptake in a subgroup of patients; however, in that study, mutational status was not reported [17]. The first proof-of-principle in a clinical study was recently published by Bahce et al. in which NCSLC patients with responsive EGFR exon 19 deleted tumors showed increased uptake of [^11^C]erlotinib when compared to patients with non-responsive EGFR wild-type tumors. Furthermore, in these patients, a metabolite analysis was performed demonstrating circa 50% intact [^11^C]erlotinib in plasma and mainly polar metabolites. This was the first study ever demonstrating the predictive potential of an EGFR TKI-PET tracer; therefore, this tracer was used as ‘gold standard’ reference in this study [18].

Afatinib (Giotrif/Gilotrif®, Boehringer Ingelheim, Ingelheim, Germany; **2**, Figure 1) is a second-generation irreversible 4-anilinoquinazoline inhibitor of EGFR [19]. By virtue of its Michael acceptor moiety, it is an irreversible inhibitor, which acts by covalent binding to a cysteine residue in the ATP binding domain of EGFR (Cys 797), HER2 (Cys 805), and ERBB4 (Cys 803) [19,20]. It can be hypothesized that the covalent binding of afatinib to EGFR could result in longer retention in the tumor as compared to the reversible TKI erlotinib and could thus provide a PET probe with improved tumor retention. Afatinib has recently gained approval for the treatment of patients with metastatic NSCLC whose tumors harbor common EGFR-activating mutations [11].

We recently reported the fluorine-18 labeling of afatinib and initial preclinical evaluation [21]. These studies demonstrated excellent *in vivo* stability of the tracer, with over 80% of intact [^18^F]afatinib present 45 min post injection (PI) in the blood plasma. Uptake in NSCLC xenografted mice was also observed. These achievements now allow for the first time the direct comparison of the tumor-targeting potential of the first-generation reversible TKI [^11^C]erlotinib and the second-generation irreversible TKI [^18^F]afatinib, both approved for the treatment of NSCLC. The aim of this study was to determine whether irreversible TKIs have improved tumor-targeting properties and kinetics and to investigate the influence of drug efflux transporters on the tumor uptake kinetics of these compounds.

## Methods

### Cell lines and reagents

Human lung cancer cell lines A549, H1975, and HCC827 were obtained from the American Type Culture Collection. Erlotinib was obtained from Sequioa Research Products (Pangbourne, UK), and afatinib was obtained from Axxon Medchem (Groningen, The Netherlands).

### Xenografts

Female athymic nude mice (20 to 25 g) (Harlan Laboratories, Horst, The Netherlands) were housed in sterile cages under standard conditions (24°C, 60% relative humidity, 12-h light/dark cycles) and provided with water and food *ad libitum*. All reported studies were performed according to the national regulation and approved by the local animal experiments ethical committee (VU University Medical Center animal experimentation ethics committee). Subcutaneous tumors were induced by inoculating approximately 2 × 10^6^ cells of the A549, H1975, or HCC827 cell lines on the left flank. Approximately 1 to 2 weeks after tumor cell inoculation, the tumors were of suitable size (100 to 200 mm^3^).

### Sequencing of xenografts

EGFR mutation analysis was performed on DNA isolated from xenografts using high-resolution melting followed by cycle sequencing of PCR products displaying a suspect melting profile, as described before [22].

### Immunohistochemical staining

Cryosections of frozen xenografts (A549, H1975, and HCC827) were immunostained for the assessment of EGFR and permeability glycoprotein (P-gp) expression. Antibodies were diluted in phosphate-buffered saline (PBS) with 1% bovine serum albumin. EGFR was stained with cetuximab (Merck & Company, Whitehouse Station, NJ, USA) and P-gp with rabbit polyclonal anti-P-gp (AB103477, ITK Diagnostics BV, Uithoorn, The Netherlands). As secondary antibodies, rabbit anti-human horseradish peroxidase (P0214, Dako Denmark A/S, Glostrup, Denmark) or swine anti-rabbit horseradish peroxidase (P0217, Dako) were used. Cryosections (5 μm) of fresh frozen (tumor) tissue were air-dried and subsequently fixed with 2% paraformaldehyde in PBS for 10 min. The sections were blocked with normal rabbit serum (in case of cetuximab) or with normal swine serum (in case of anti-P-gp) and subsequently stained with cetuximab 10 μg/mL (EGFR) or anti-P-gp 5 μg/mL. Color development was performed with diaminobenzidine (DAB) and counterstaining was done with hematoxylin.

### Synthesis of radiotracers

**Scheme 1:**
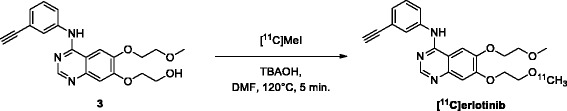
**Radiosynthesis of [** ^**11**^ **C]erlotinib.** TBAOH, tetrabutylammonium hydroxide; DMF, dimethylformamide.

[^11^C]erlotinib [18] was synthesized as previously described (Scheme 1). Briefly, cyclotron-produced [^11^C]CO_2_ was reduced using LiAlH_4_ to obtain [^11^C]CH_3_OLi and the latter was subsequently halogenated using hydrogen iodide. The obtained [^11^C]MeI was distilled to a new vessel containing the hydroxyl labeling precursor **3** (1.0 mg, 2.5 μmol) and tetrabutylammonium hydroxide (TBAOH, 5 M aqueous solution, 2 μL, 10 μmol) as a supporting base in acetonitrile (250 μL). The alkylation is performed for 5 min at 120°C. The product was isolated by semi-preparative high-performance liquid chromatography (HPLC), carried out on a Jasco PU-2089 pump (Jasco Inc. Easton, MD, USA) equipped with a SymmetryPrep C-18 (Waters Corporation, Milford, MA, USA; 7 μm, 300 mm × 7.8 mm) using MeCN/25 mM sodium phosphate buffer pH = 3.5 (30:70, *v*/*v*) as eluent at a flow rate of 4.3 mL/min, a Jasco UV1575 UV detector (*λ =* 254 nm) and a custom-made radioactivity detector. Chromatograms were acquired using ChromNAV software (version 1.14.01, Jasco). The collected fraction of the preparative HPLC purification containing the product was diluted with 40 mL of aqueous NaOH (1 mM), and the total mixture was passed over a tC18 Plus Sep-Pak cartridge. The cartridge was then washed with 20 mL of sterile water after which the product was eluted from the cartridge with 1.0 mL of sterile 96% ethanol. The ethanol was diluted to 10 vol.% with formulation solution (7.09 mM NaH_2_PO_4_ in 0.9% NaCl, *w*/*v* in water, pH 5.2), and the complete solution was filtered over a Millex-GV 0.22-μm filter into a sterile 20 mL capped vial to provide a final solution of 10% ethanol in saline (containing 7.09 mM NaH_2_PO_4_) containing [^11^C]erlotinib in >99% radiochemical purity as an intravenous (IV) injectable solution in a total synthesis time of less than 30 min (from end of isotope production) in high specific activity (287 ± 63 GBq/μmol) and in 13.1% ± 3.7% yield (corrected for decay, up to 3 GBq isolated).

**Scheme 2:**
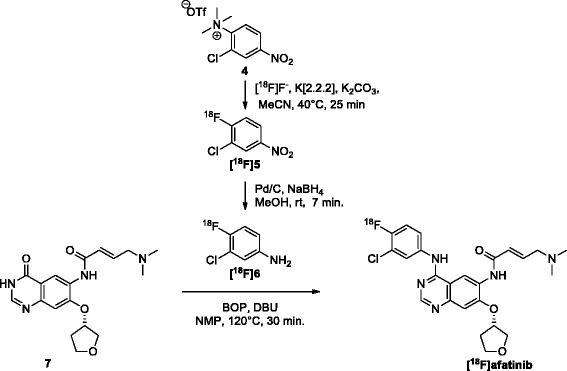
**Radiosynthesis of [** ^**18**^ **F]6 and subsequent condensation towards [** ^**18**^ **F]afatinib.** MeCN, acetonitrile; MeOH, methanol; DBU, 1,8-diazabicyclo[5.4.0]undec-7-een; NMP, *N*-methylpyrrolidone.

[^18^F]afatinib [21] was synthesized as previously reported (Scheme 2). Briefly, cyclotron-produced [^18^F]fluoride was azeotropically dried with acetonitrile/water (9/1, *v*/*v*) containing K[2.2.2] (4,7,13,16,21,24-hexaoxa-1,10-diazabicyclo[8.8.8]hexacosane, 13.0 mg, 34.6 μmol) and potassium carbonate (2.0 mg, 15 μmol). To the dried residue was added a solution of 3-chloro-4-trimethylammonium-nitrobenzene triflate (**4**, 3.0 mg, 14 μmol) in acetonitrile (0.7 mL), and the mixture was allowed to react for 25 min at 40°C. After the reaction mixture was quenched with water (7 mL), 3-chloro-4-[^18^F]fluoronitrobenzene ([^18^F]**5**) was trapped on a tC18 Plus Sep-Pak, rinsed with water (10 mL), and subsequently eluted with MeOH (1.5 mL) into a screw cap reaction vessel containing palladium on activated carbon (10%, 3 mg) and sodium borohydride (10.0 mg, 264 μmol). The reduction was carried out for 7 min at 20°C upon which the reaction was quenched by the addition of concentrated hydrochloric acid (37%, 0.1 mL). The thus obtained mixture was passed through a syringe filter (Millex LCR PTFE 0.45 μM/25 mm; Millipore Corporation, Darmstadt, Germany) into a new screw cap reaction vessel. The volatiles were removed *in vacuo* and under a helium flow (100 mL/min) at elevated temperatures (90°C for 5 min and 120°C for 2 min) to obtain the dry 3-chloro-4-[^18^F]fluoroaniline-HCl salt ([^18^F]**6**). The product was dissolved in *N*-methylpyrrolidine (NMP, 0.5 mL) and to this solution was added a solution of (*S*,*E*)-4-(dimethylamino)-*N*-(4-oxo-7-((tetrahydrofuran-3-yl)oxy)-3,4-dihydroquinazolin-6-yl)but-2-enamide (**7**, 2 mg, 6 μmol), benzotriazole-1-yl-oxy-tris-(dimethylamino)-phosphonium hexafluorophosphate (BOP), 5.5 mg, 12 μmol, and 1,8-diazabicyclo[5.4.0]undec-7-een, 2.7 μL (DBU), 18 μmol, in anhydrous NMP (0.5 mL), which was dissolved 15 min prior to addition. The obtained mixture was heated to 120°C for 30 min after which it was cooled to 20°C. The product was isolated by semi-preparative HPLC, carried out on a Jasco PU-2089 pump equipped with a C18 Alltima column (Grace, 5 μm, 250 mm × 10 mm; Crawford Scientific, Columbia, MD, USA) using MeCN/H_2_O/DiPA (40:60:0.2, *v*/*v*/*v*) as eluent at a flow rate of 4 mL/min, a Jasco UV1575 UV detector (*λ =* 254 nm), and a custom-made radioactivity detector. Chromatograms were acquired using ChromNAV software (version 1.14.01, Jasco). The collected fraction of the preparative HPLC purification containing the product was diluted with 50 mL of water and the total mixture was passed over a tC18 Plus Sep-Pak cartridge. The cartridge was then washed with 20 mL of sterile water after which the product was eluted from the cartridge with 1.0 mL of sterile 96% ethanol. The ethanol was diluted to 10 vol.% with formulation solution (7.09 mM NaH_2_PO_4_ in 0.9% NaCl, *w*/*v* in water, pH 5.2), and the complete solution was filtered over a Millex-GV 0.22-μm filter into a sterile 20 mL capped vial. In this way, a final IV injectable solution was provided of 10% ethanol in saline (containing 7.09 mM NaH_2_PO_4_) containing [^18^F]afatinib obtained at >98% radiochemical purity, in a total synthesis time of less than 120 min (from end of isotope production), at a high specific activity (287 ± 63 GBq/μmol), and in 17.0% ± 2.5% yield (corrected for decay, up to 3.5 GBq isolated).

### PET imaging

Dynamic PET imaging was performed on three cancer xenograft lines (A549, H1975, and HCC827) in nude mice. Each mouse (*n* = 3) carried one tumor, which was located on the left flank. Imaging was performed for a duration of 90 ([^11^C]erlotinib) or 120 min ([^18^F]afatinib) using a double-LSO/LYSO-layer high-resolution research tomograph (HRRT; CTI/Siemens, Knoxville, TN, USA). The mice were anesthetized with 4% and 2% isoflurane in 1 L/min oxygen for induction and maintenance, respectively. First, for attenuation and scatter correction, a transmission scan was acquired using a 740-MBq two-dimensional (2D) fan-collimated ^137^Cs (662 keV) moving point source. Next, a dynamic emission scan was acquired immediately following administration (IV ocular plexus) of 8 to 10 MBq [^11^C]erlotinib (223 ± 38 GBq/μmol) or 4 to 6 MBq [^18^F]afatinib (287 ± 63 GBq/μmol) to each animal. Positron emission scans were acquired in list mode and rebinned into the following frame sequence: 10 × 60 s, 4 × 300 s, and 9 × 600 s. After the TKI scan was finished, [^18^F]FDG was administered (IV ocular plexus) to the mice followed by scanning for another 60 min. Following corrections for decay, dead time, scatter, and randoms, the scans were reconstructed using an iterative 3D-ordered subsets weighted least-squares analysis (3D-OSWLS). The point source resolution varied across the field of view from approximately 2.3- to 3.2-mm full width at half maximum in the transaxial direction and from 2.5 to 3.4 mm in the axial direction. Post-filtering was not performed after reconstruction. The PET images were analyzed using the freely available AMIDE software version 0.9.2 (a medical imaging data examiner). A box was drawn over the complete animal to obtain the image-derived percentage injected dose per gram (%ID/g). Regions of interest (ROIs) containing the tumor tissue as well as a reference area, which was drawn in the opposite flank of the animal containing exactly the same tissue only devoid of tumor cells, were drawn using the [^18^F]FDG data; the tumor region was defined as FDG positive voxels of the tumor. Subsequently, the corresponding images obtained with [^11^C]erlotinib or [^18^F]afatinib were overlayed. A time-activity-curve (TAC) was plotted for both the tumor as well as the reference area. The images were smoothed using a Gaussian filter (2 mm).

For PET imaging studies using mass amounts, afatinib (1.0 mg) was dissolved in DMSO (1.0 mL) and diluted with formulation solution (7.09 mM NaH_2_PO_4_ in 0.9% NaCl, *w*/*v* in water, pH 5.2) to the appropriate concentrations (40, 120, 400, and 1,200 nM), and after addition of 50 μL [^18^F]afatinib to 50 μL of these solutions, they were injected as a IV bolus.

For the P-gp blocking experiments, tariquidar (7.5 mg/mL, Azatrius Pharmaceuticals Pvt. Ltd. Mumbai, India) was diluted to 3.75 mg/mL with saline for injection. In the blocking experiments, tariquidar (15 mg/kg) was administered IV 20 min prior to tracer injection [23].

### Statistical analysis

Statistical analysis on tumor-to-background ratios was performed using Graphpad PRISM (v 5.02, Graphpad Software Inc). The concentration of activity in the tumor (%ID/g, *n* = 3 per group) was compared to the concentration of activity in the reference tissue using a one-tailed Student’s *t*-test for paired data.

## Results and discussion

### Radiochemistry

The synthesis of [^11^C]erlotinib has been described previously and involves a straightforward alkylation employing [^11^C]MeI on a terminal alcohol (**3**) depicted in Scheme 1, providing [^11^C]erlotinib in high radiochemical yields (up to 4 GBq) and high specific activity (287 ± 63 GBq/μmol) [18].

The synthesis of [^18^F]afatinib (Scheme 2) was recently published by our group and involves a BOP-mediated coupling of 3-chloro-4-[^18^F]fluoroaniline ([^18^F]**6**) with **7** as a key step [21]. The synthesis of [^18^F]afatinib starts with nucleophilic fluorination on **4** with ^18^F^−^/K[2.2.2] (kryptofix, 4,7,13,16,21,24-hexaoxa-1,10-diazabicyclo[8.8.8]hexacosane) in the presence of K_2_CO_3_, for 25 min. After a solid phase extraction, the resulting 3-chloro-4[^18^F]fluoronitrobenzene ([^18^F]**5**) is subjected to a reduction using sodium borohydride in the presence of palladium on carbon, thus furnishing the desired 3-chloro-4[^18^F]fluoroaniline ([^18^F]**6**). The final step is a BOP-mediated condensation of aniline **6** with **7** to provide [^18^F]afatinib in high yields (up to 3.5 GBq) and high specific activity (223 ± 38 GBq/μmol) after HPLC purification. Initial *in vivo* stability as well as *ex vivo* biodistribution studies demonstrated excellent *in vivo* stability of the tracer and uptake in NSCLC xenografted mice, although no large differences with regard to uptake between wild-type (WT) and the exon 19 deleted mutant of EGFR were observed in *ex vivo* biodistribution studies, therefore necessitating further PET studies described in this work [21].

### Xenograft characterization

Three NSCLC cell lines were selected for the generation of xenografts and the *in vivo* evaluation of [^11^C]erlotinib and [^18^F]afatinib, each expressing a specific EGFR mutation and thus providing a representative overview of mutations found in clinical cases of NSCLC [24]. Firstly, an insensitive reference cell line which expresses EGFR wild type was selected (A549). Next, a cell line (H1975) which expresses a double mutant of EGFR (L858R/T790M); the first being one of the common sensitizing point mutation in exon 21 (L858R) and the second a mutation associated with acquired resistance to erlotinib therapy (T790M in exon 20). Finally, a cell line was selected which is highly sensitive to TKI treatment, namely the HCC827 cell line which harbors a deletion in exon 19 (delE746-A750). The sensitivity of these cell lines towards the two inhibitors has been clearly described in literature [19,25,26]. The results of *in vitro* studies and *in vivo* xenograft experiments demonstrated excellent efficacy of both inhibitors. The double mutant H1975 cells were shown to be resistant to treatment with erlotinib but showed a reduction in tumor growth rate *in vivo* upon afatinib treatment, although significantly less than HCC827. Finally, neither erlotinib nor afatinib showed any *in vitro* or *in vivo* efficacy to the wild-type A549 cells and xenografts, which despite expressing EGFR are not dependent on the ErbB signaling network for proliferation*.*

**Figure 2:**
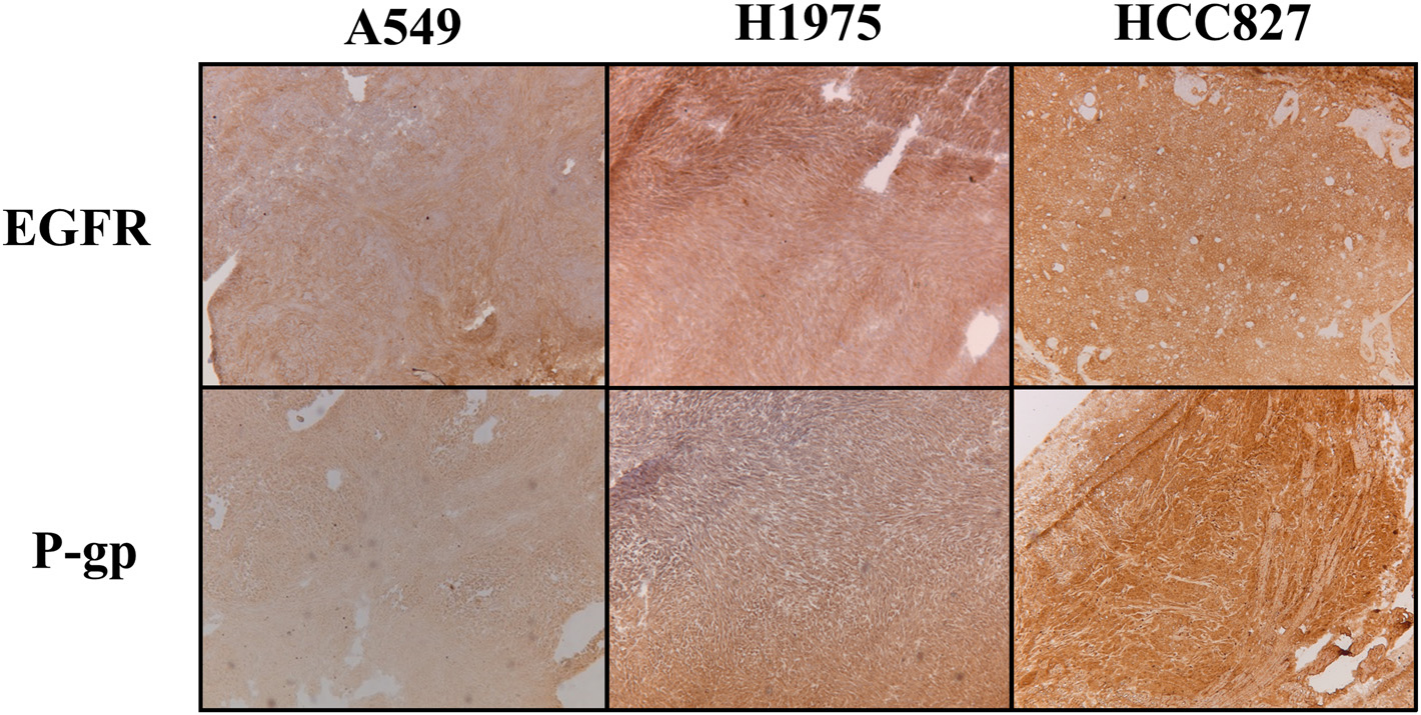
**Immunohistochemical staining of NSCLC xenograft lines as used in PET studies.** Images depicted at 5× magnification. Mutations: A549 (wild type), H1975 (L858R/T790M), and HCC827 (exon 19 deletion).

To fully characterize these cell lines before the start of PET imaging studies, they were xenografted onto athymic nu/nu mice and immunohistochemistry and sequencing were performed on the xenograft material. EGFR expression was found in all lines (Figure 2) and although immunohistochemistry is a semi-quantitative technique, staining appeared most intense in HCC827 xenografts. The EGFR mutations of the xenografts, as described before, were confirmed by sequencing. Finally, the tumors were stained for the expression of P-gp, a drug efflux transporter, for which erlotinib and afatinib are known substrates [27,28]. The staining indicated that this efflux transporter was most extensively expressed by HCC827 xenografts.

### PET imaging with [^11^C]erlotinib and [^18^F]afatinib in lung cancer xenografts

PET imaging was performed with both tracers in all three xenograft models to determine whether the irreversible inhibitor shows better tumor targeting. In order to have a proper region for the background tissue, the mice were grafted with a single tumor on the left flank. In this manner, the right flank of the animal served as reference region as this contralateral tissue is the same except that it is devoid of tumor cells. Both tumor and background were manually outlined on the basis of an additional [^18^F]FDG scan, which was performed directly after the TKI-PET scan was finished. Percentage injected dose per gram of tissue can be directly derived from these PET scans as a quantitative measure of uptake.

**Figure 3:**
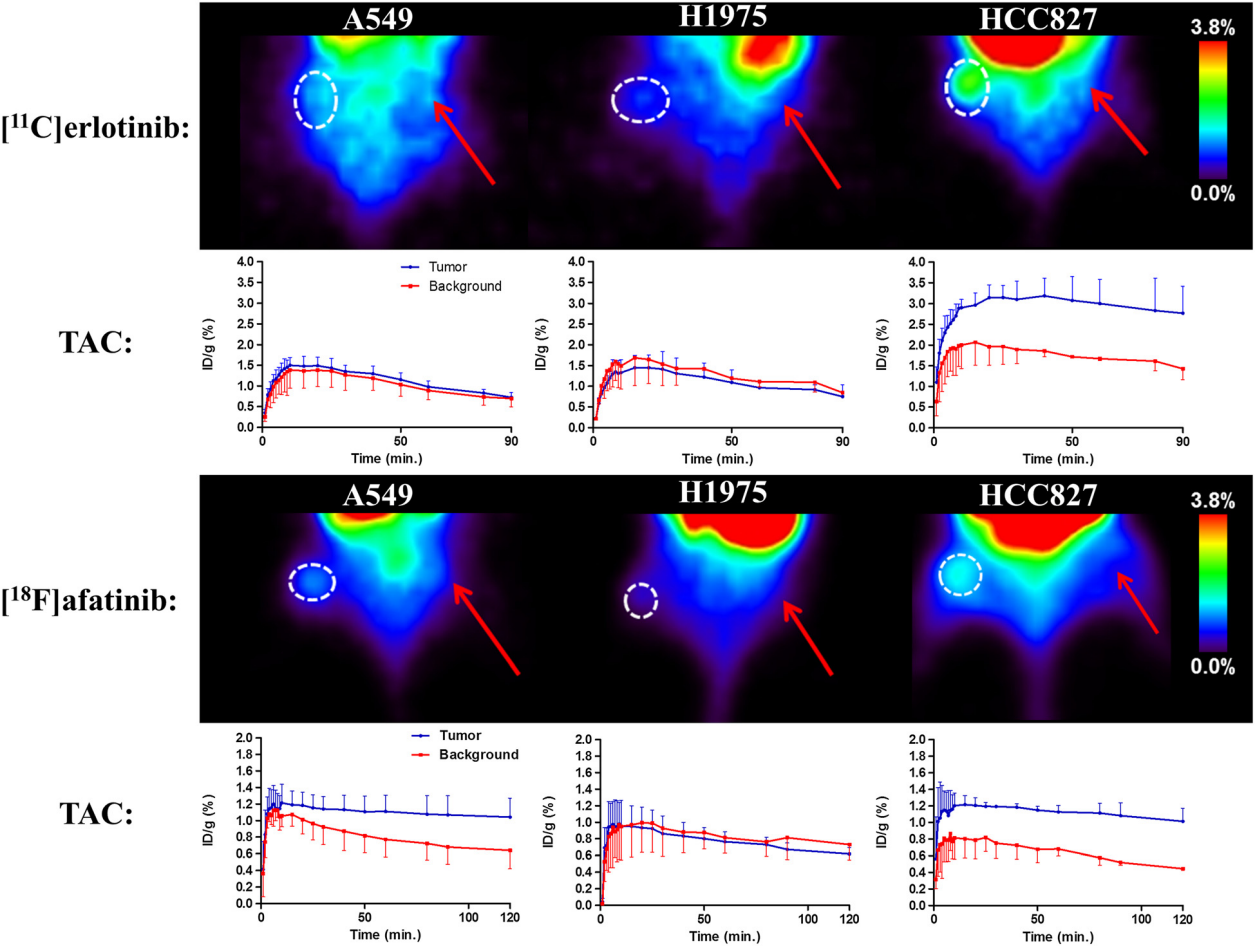
**PET images and TACs of [** ^**11**^ **C]erlotinib (top) and [** ^**18**^ **F]afatinib (bottom).** Circle indicates tumor position and arrow indicates reference tissue. [^11^C]erlotinib images summed from 60 to 90 min, [^18^F]afatinib images from 90 to 120 min. TACs are averaged over three animals. Mutations: A549 (wild type), H1975 (L858R/T790M), and HCC827 (exon 19 deletion). Tumor and background were manually outlined on the basis of an additional [^18^F]FDG scan, which was performed directly after the TKI-PET scan was finished.

The images and time-activity-curves (TACs, depicting activity concentration over time) of the PET study are shown in Figure 3, and the tumor-to-background ratios (resulting from a comparison between activity concentration in the tumor and reference tissue) are summarized in Table 1. Since [^18^F]afatinib has a longer half-life than [^11^C]erlotinib, [^18^F]afatinib could be scanned for longer time periods. However, no significant changes were observed in this later frame with regard to uptake. The images shown (Figure 3) are from the last 30 min of the scan (60 to 90 min for [^11^C]erlotinib and 90 to 120 min for [^18^F]afatinib), representing the highest tumor-to-background ratios. High uptake is observed in the liver and kidneys for both tracers, which is common for IV-administered small molecular PET tracers as these organs represent the major excretion and catabolic routes. The most striking difference between the two tracers is that [^11^C]erlotinib displays significantly slower kinetics when compared to [^18^F]afatinib, irrespective of the xenograft line studied. Indeed, [^11^C]erlotinib (reversible inhibitor) reaches its peak uptake in the tumor (HCC827) at 25 min of 3.2 ± 0.3%ID/g PI, whereas [^18^F]afatinib (irreversible inhibitor) activity concentration in the tumor (HCC827) is already at a maximum of 1.2 ± 0.2%ID/g at 10 min PI, indicating faster kinetics and/or clearance (Figure 3). This results in a higher activity concentration in the case of [^11^C]erlotinib in all tissues of interest (tumor and background) when compared to [^18^F]afatinib.

**Table 1:** **Summary of tumor-to-background ratios**

**Cell line**	**Number**	**Tumor-to-background [** ^**11**^ **C]erlotinib**	**Number**	**Tumor-to-background [** ^**18**^ **F]afatinib**
Unblocked				
A549	1	1.0 ± 0.3	4	1.5 ± 0.3*
H1975	2	0.9 ± 0.3	5	0.8 ± 0.2
HCC827	3	1.9 ± 0.5*	6	2.3 ± 0.4*
Tariquidar blocked (15 mg/kg)				
A549	7	1.2 ± 0.3	10	1.3 ± 0.3*
H1975	8	1.0 ± 0.2	11	0.8 ± 0.2
HCC827	9	1.8 ± 0.3*	12	1.9 ± 0.4*

Comparison of tumor-to-background ratios in non-blocked (1 to 6) and blocked situation (7 to 12) ratios determined in the last frame of the scans (60 to 90 min for [^11^C]erlotinib and 90 to 120 min for [^18^F]afatinib) and averaged over three animals. P-gp blocking was performed with 15 mg/kg tariquidar. *Tumor-to-background is statistically significant (*P* < 0.05, Student’s *t*-test for paired data). Mutations: A549 (wild type), H1975 (L858R/T790M), and HCC827 (exon 19 deletion).

Regarding the general imaging properties, both tracers appear to have a similar uptake pattern across the various xenograft lines with their distinguished mutational status (Figure 3). In xenografts expressing wild-type EGFR (A549), [^11^C]erlotinib does not show any selective accumulation which is comparable with previous literature reported on this tracer [16]. [^18^F]afatinib demonstrates a modest, yet statistically significant uptake in A549 cells (Table 1, entry 4).

In the double mutant xenografts (H1975), which harbor a sensitizing mutation (L858R), and an acquired resistance mutation (T790M), no selective uptake was observed for either tracer (Table 1, entries 2 and 5). This result is in good accordance with the efficacy of erlotinib, as it shows no therapeutic effect in this xenograft model. However, afatinib showed a modest but significant reduction in tumor growth rate of H1975 xenografts [20,25], whereas no significant tumor uptake of [^18^F]afatinib was observed (Figure 3).

Both [^11^C]erlotinib and [^18^F]afatinib demonstrate best uptake in the HCC827 xenografts. [^18^F]afatinib demonstrates a slightly higher tumor-to-background ratio than [^11^C]erlotinib (Table 1, entries 3 and 6). This data demonstrates the ability of TKI-PET to image TKI uptake in these tumors in an effective manner. It was previously demonstrated that uptake of [^11^C]erlotinib in HCC827 xenografts could be blocked by the addition of unlabeled erlotinib [29]. To ascertain whether imaging should be performed at high specific activity in the case of [^18^F]afatinib (4 to 6 MBq, 0.017 to 0.026 nmol of afatinib), PET imaging with co-administration of unlabeled afatinib (100 to 6,000 ng) in a bolus IV injection with the tracer was performed in the HCC827 xenograft line. This demonstrated that the uptake of the tracer was blocked to the background level with a 100-ng addition of afatinib (Table 2).

**Table 2:** **Blocking of [** ^**18**^ **F]afatinib uptake in HCC827 xenografts**

**Added dose of afatinib (ng)**	**Tumor-to-background ratio**
0	2.3 ± 0.4*
100	1.1 ± 0.4
300	1.0 ± 0.3
1,000	0.9 ± 0.2
3,000	0.9 ± 0.3

Tumor-to-background ratio after a bolus injection of [^18^F]afatinib with added isotopically unmodified afatinib. Ratios determined in the last frame of the scans (90 to 120 min) and averaged over three animals.*Tumor-to-background is statistically significant (*P* < 0.05, Student’s *t*-test for paired data). HCC827 xenografts harbor an exon 19 deleted variant of EGFR.

Several distinct observations can be made from the results described above. Firstly, the absolute uptake (%ID/g, Figure 3) of [^18^F]afatinib in HCC827 xenografts is lower in comparison with uptake of [^11^C]erlotinib, where this was expected to be at least comparable, based on the respective affinities of the compounds for EGFR. Immunohistochemistry demonstrated that HCC827 cells have a high P-gp expression and thus, there is a possibility that drug efflux is playing a role in this xenograft line. However, differences in biophysical properties such as lipophilicity, basicity, or passive permeation may also influence the apparent uptake of these compounds. Secondly, when comparing the reversible [^11^C]erlotinib with irreversible [^18^F]afatinib, no significant differences are observed with regard to the kinetic profile displayed in the TACs for this xenograft model (Figure 3). Thirdly, no differential uptake compared to the background of [^18^F]afatinib was observed in H1975 tumors despite the fact that afatinib is therapeutically effective in this xenograft line (Figure 3). Finally, in the wild-type xenograft line (A549), no uptake of [^11^C]erlotinib was observed, while [^18^F]afatinib did show modest uptake (Figure 3). This could be due to differences in retention which might be attributed to the biophysical differences between the two tracers, the ability of [^18^F]afatinib to bind covalently to additional targets (HER2 and ERBB4 next to EGFR) or differences in affinity for efflux transporters. In an attempt to gain further insight into the role of P-gp in PET tracer uptake, an imaging study was performed in the presence of an efflux transporter inhibitor.

### Influence of drug efflux transporter on tracer uptake

P-glycoprotein (or multidrug resistance protein 1; MDR1) is an ATP-dependent efflux pump that is responsible for the transport of foreign substrates out of the cells and thereby serves as a defense mechanism against these substrates. P-gp expression was previously observed for H1975 and HCC827 using immunohistochemistry and was higher in the latter, indicating that P-gp inhibition could result in an increased uptake of the PET tracers [27,28].

**Figure 4:**
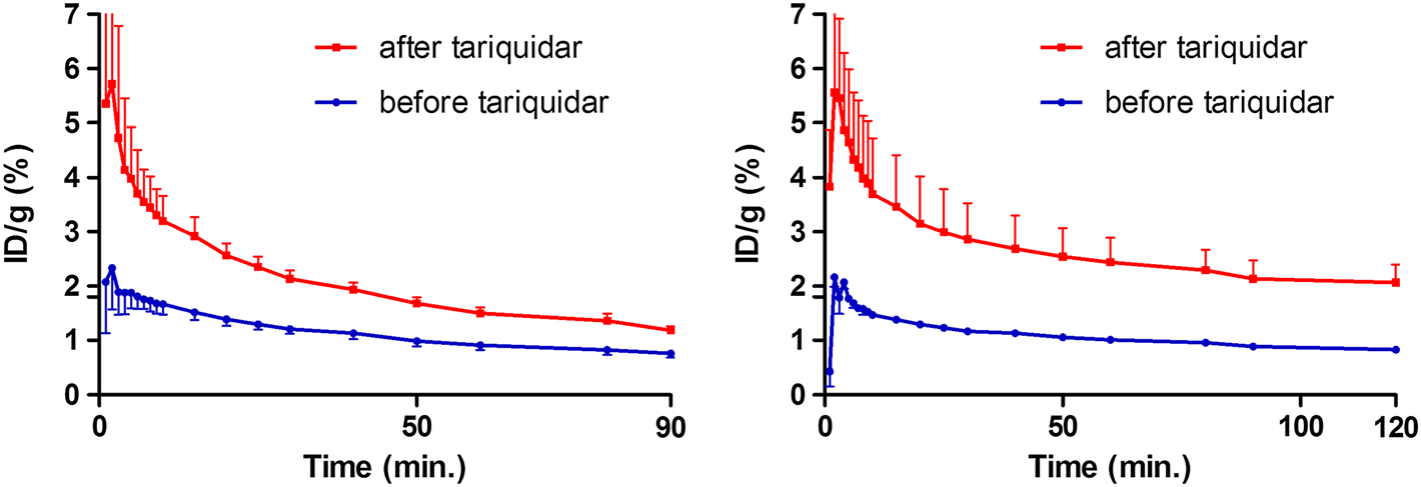
**Whole brain TACs of [** ^**11**^ **C]erlotinib (left) and [** ^**18**^ **F]afatinib (right) before and after tariquidar pre-treatment.** TACs are averaged over three animals.

Dynamic PET imaging was performed while blocking P-gp using tariquidar (15 mg/kg). Tariquidar was successfully applied in previous PET studies to block P-gp [23,30], although it was recently demonstrated that is also an inhibitor of breast cancer resistance protein BCRP, another ATP-binding cassette (ABC) family efflux transporter [31]. Tracer uptake in brain was also monitored as the blood-brain barrier has, among other efflux transporters, high P-gp expression. Pre-treatment with tariquidar resulted in an increased uptake of both [^11^C]erlotinib and [^18^F]afatinib in the brain (Figure 4), thus showing that biodistribution of both compounds (both P-gp substrates) can be influenced by P-gp blockers.

**Figure 5:**
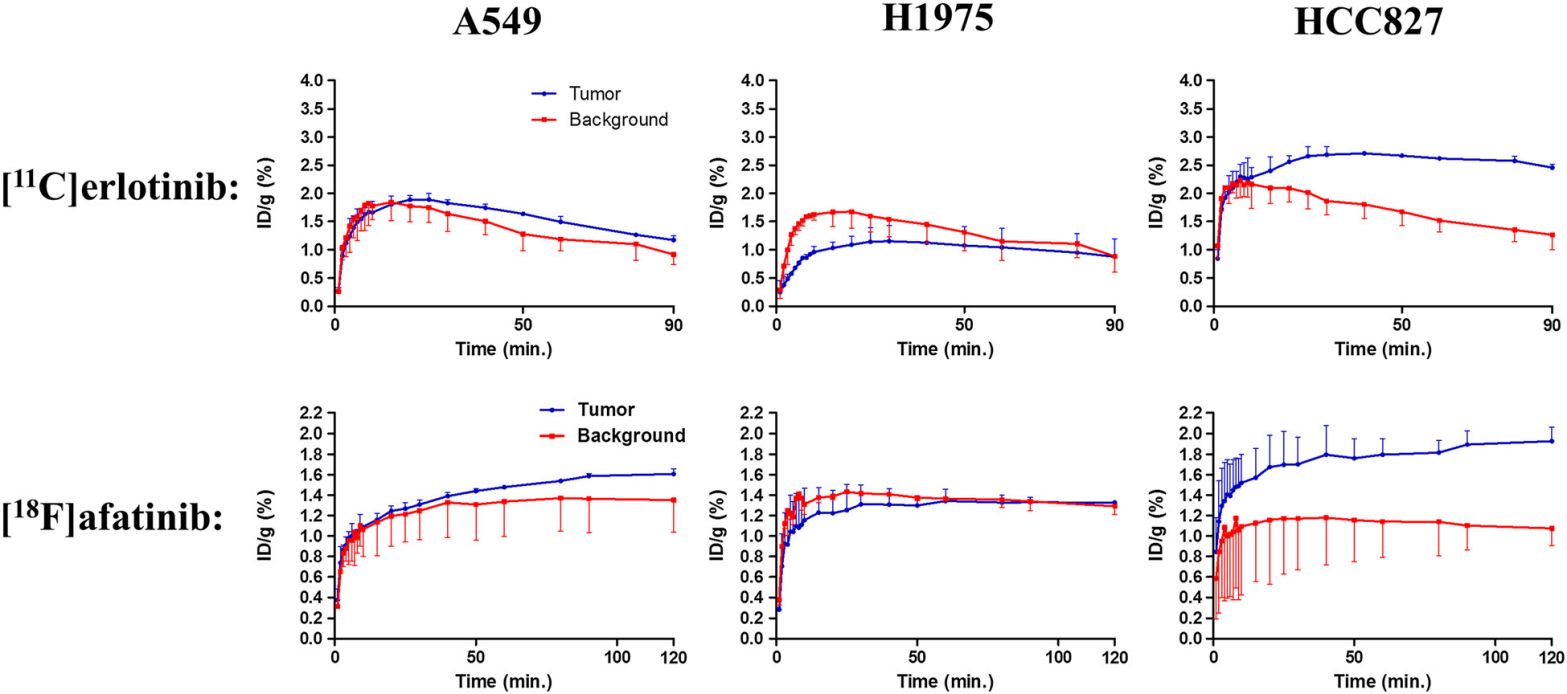
**TACs of [** ^**11**^ **C]erlotinib (top) and [** ^**18**^ **F]afatinib (bottom) after tariquidar treatment.** TACs are averaged over three animals. Mutations: A549 (wild type), H1975 (L858R/T790M), and HCC827 (exon 19 deletion). Tumor and background were manually outlined on the basis of an additional [^18^F]FDG scan, which was performed directly after the TKI-PET scan was finished.

The TACs of the pre-blocked mice bearing the same tumor models as in the non-blocked condition are depicted in Figure 5. In the wild-type xenograft (A549), a minor increase of [^11^C]erlotinib tumor accumulation was observed which is also reflected in the tumor-to-background ratio (Table 1, entry 7), although this was not found to be statistically significant. In this xenograft model, a higher uptake was observed after treatment with tariquidar in both the tumor and the background (compare Figure 5 with Figure 3). [^18^F]afatinib also demonstrates a higher absolute uptake of activity (%ID/g) in the tumor and background and no washout was observed in either tissues. Apparently, the blocking of the efflux transporter systems with tariquidar resulted in better tissue trapping of [^18^F]afatinib (Figure 5).

The double mutant (H1975) showed no uptake in the tumor upon P-gp inhibition for [^11^C]erlotinib (Table 1, entry 8) similar to the unblocked situation (Table 1, entry 2). For [^18^F]afatinib, a higher activity concentration was observed (Figure 5), although the tumor-to-background ratio remained similar to the non-blocked situation (Table 1, entries 5 and 11).

Finally, in the HCC827 xenograft, no differences were observed for [^11^C]erlotinib in tumor-to-background ratio (Table 1, entries 3 and 9). In the case of [^18^F]afatinib, a substantial increase of activity concentration was observed (maximal activity concentration 1.9 ± 0.1%ID/g vs 1.2 ± 0.2%ID/g, Figure 3), although this did not lead to higher tumor-to-background ratio due to a similar increase in the background tissue. It does, however, indicate that [^18^F]afatinib is influenced by efflux transporters to a larger extent than [^11^C]erlotinib. This is also observed in the kinetics of [^18^F]afatinib binding, as these are significantly affected. Retention of activity was observed in the HCC827 model in the TAC (Figure 5). This observation is in line with the irreversible mode of binding of afatinib.

An important observation from the blocking study is a moderate increase of activity in all tissues studied for [^18^F]afatinib (Figure 5). This may be caused by an increase of activity in the blood pool, under P-gp blocking conditions, and hence increased delivery of [^18^F]afatinib to tissues. This can be explained by the inverse function of P-gp in the intestinal lumen where it normally extracts xenobiotics from the blood [32]. This extraction may be reduced due to the blocking of P-g by tariquidar, resulting in a higher concentration of [^18^F]afatinib in the blood. The relatively large increase of [^18^F]afatinib accumulation in the HCC827 tumors indicates that efflux transporters play a significant role in the apparent uptake or efflux of afatinib from these tumors. In the EGFR wild-type xenograft A549, showing low P-gp expression, this difference was much less pronounced (Figure 5).

Interestingly the [^18^F]afatinib TACs in the A549 and HCC827 xenografts demonstrate an irreversible character with regard to uptake of [^18^F]afatinib under P-gp blocking conditions (Figure 5). It might well be that efflux by P-gp in the non-blocked situation is relatively fast and quicker than the irreversible binding, resulting in what appears to be reversible kinetics. This effect is not observed for [^11^C]erlotinib and is in line with a reversible mode of binding. The H1975 xenograft demonstrated no uptake in the blocked or non-blocked situation, which was unexpected on the basis of affinity.

The irreversible binding of afatinib to EGFR-WT and EGFR-T790M was demonstrated *in vitro* by Solca et al. in several experiments, including mass spectrometry and X-ray crystallography of afatinib bound to EGFR-T790M, where a covalent bond between afatinib and EGFR-T790M was demonstrated [19]. The results from the current PET studies suggest that the *in vivo* tumor uptake of [^11^C]erlotinib and [^18^F]afatinib is influenced by P-gp expression levels and tracer uptake is not completely predictive for the therapeutic efficacy in the H1975 xenograft line. One of the reasons might be that in the current study, [^11^C]erlotinib and [^18^F]afatinib are administered IV at a tracer dose (μg/kg), while efficacy is tested after oral administration at a therapeutic dose (mg/kg). This justifies further characterization of [^11^C]erlotinib and [^18^F]afatinib in PET studies under therapeutic dosage conditions.

## Conclusions

Both [^11^C]erlotinib and [^18^F]afatinib are useful TKI-PET tracers for imaging treatment-sensitive xenografts harboring exon 19 deletion mutations in EGFR. The good tumor-to-background ratios could in the future be used in clinical decision making for both tracers. The difference between a reversible and irreversible inhibitor could not be demonstrated within a standard PET imaging situation as both tracers showed similar tumor uptake kinetics. However, when the drug efflux transporter P-gp is blocked, increased tumor uptake is observed and under those conditions [^18^F]afatinib reveals different kinetics in the HCC827 model suggestive of irreversible binding. This shows that preclinical TKI-PET imaging can be used to compare tumor-targeting properties and tumor kinetics of TKIs, making it a valuable tool for drug design and selection.

## Abbreviations

%ID/g: percentage injected dose per gram (of tissue)
ABC: ATP-binding cassette
ATP: adenosine triphosphate
BOP: (benzotriazol-1-yloxy)tris(dimethylamino)phosphonium hexafluorophosphate
DAB: diaminobenzidine
DBU: 1,8-diazabicyclo[5.4.0]undec-7-een
DMF: dimethylformamide
DNA: deoxyribonucleic acid
EGFR: epidermal growth factor receptor
FDG: fluorodeoxyglucose
g: gram
h: hour
HPLC: high-performance liquid chromatography
IV: intravenous
K[2.2.2]: 4,7,13,16,21,24-hexaoxa-1,10-diazabicyclo[8.8.8]hexacosane
MDR1: multidrug resistance protein 1
MeI: methyliodide
MeOH: methanol
min: minutes
NSCLC: non-small cell lung cancer
PI: post injection
PBS: phosphate-buffered saline
PCR: polymerase chain reaction
PET: positron emission tomography
P-gp: permeability glycoprotein
ROI: region of interest
RTK: receptor tyrosine kinase
TAC: time-activity-curve
TBAOH: tetrabutylammonium hydroxide
TKI: tyrosine kinase inhibitor
TKI-PET: positron emission tomography with tyrosine kinase inhibitors
VEGFR: vascular endothelial growth factor receptor
WT: wild-type
